# Transcriptome-scale analysis uncovers conserved residues in the hydrophobic core of the bacterial RNA chaperone Hfq required for small regulatory RNA stability

**DOI:** 10.1093/nar/gkaf019

**Published:** 2025-01-27

**Authors:** Josh McQuail, Miroslav Krepl, Kai Katsuya-Gaviria, Aline Tabib-Salazar, Lynn Burchell, Thorsten Bischler, Tom Gräfenhan, Paul Brear, Jiří Šponer, Ben F Luisi, Sivaramesh Wigneshweraraj

**Affiliations:** Centre for Bacterial Resistance Biology, Imperial College London, LondonSW7 2AZ, United Kingdom; Institute of Biophysics of the Czech Academy of Sciences, Kralovopolska 135, Brno612 00, Czech Republic; Department of Biochemistry, University of Cambridge, CambridgeCB2 1GA, United Kingdom; Centre for Bacterial Resistance Biology, Imperial College London, LondonSW7 2AZ, United Kingdom; Centre for Bacterial Resistance Biology, Imperial College London, LondonSW7 2AZ, United Kingdom; Core Unit Systems Medicine, University of Würzburg, D-97080 Würzburg, Germany; Core Unit Systems Medicine, University of Würzburg, D-97080 Würzburg, Germany; Department of Biochemistry, University of Cambridge, CambridgeCB2 1GA, United Kingdom; Institute of Biophysics of the Czech Academy of Sciences, Kralovopolska 135, Brno612 00, Czech Republic; Department of Biochemistry, University of Cambridge, CambridgeCB2 1GA, United Kingdom; Centre for Bacterial Resistance Biology, Imperial College London, LondonSW7 2AZ, United Kingdom

## Abstract

The RNA chaperone Hfq plays crucial roles in bacterial gene expression and is a major facilitator of small regulatory RNA (sRNA) action. The toroidal architecture of the Hfq hexamer presents three well-characterized surfaces that allow it to bind sRNAs to stabilize them and engage target transcripts. Hfq-interacting sRNAs are categorized into two classes based on the surfaces they use to bind Hfq. By characterizing a systematic alanine mutant library of Hfq to identify amino acid residues that impact survival of *Escherichia coli* experiencing nitrogen (N) starvation, we corroborated the important role of the three RNA-binding surfaces for Hfq function. We uncovered two, previously uncharacterized, conserved residues, V22 and G34, in the hydrophobic core of Hfq, to have a profound impact on Hfq’s RNA-binding activity *in vivo*. Transcriptome-scale analysis revealed that V22A and G34A Hfq mutants cause widespread destabilization of both sRNA classes, to the same extent as seen in bacteria devoid of Hfq. However, the alanine substitutions at these residues resulted in only modest alteration in stability and structure of Hfq. We propose that V22 and G34 have impact on Hfq function, especially critical under cellular conditions when there is an increased demand for Hfq, such as N starvation.

## Graphical Abstract

**Graphical Abstract:**
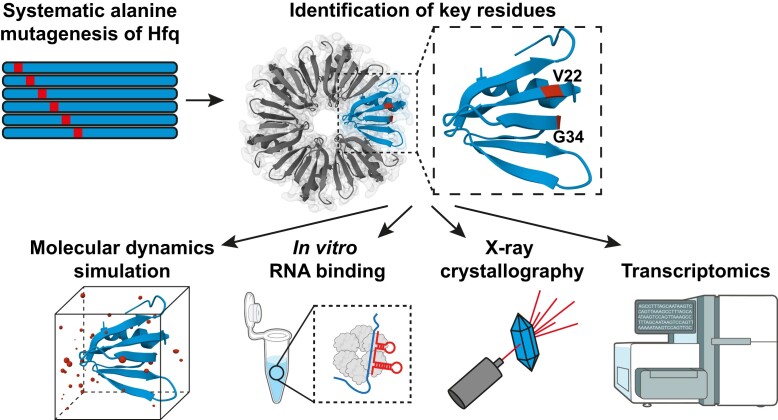

## Introduction

The post-transcriptional regulation of RNA stability and translational efficiency allows enormous flexibility in the control of genetic information in bacteria. Small non-coding regulatory RNA molecules, called sRNAs, play a pivotal role in post-transcriptional regulation of messenger RNA (mRNA). sRNAs determine whether the targeted mRNAs are destined for translation, translational repression, degradation, or stabilization. A core component of post-transcriptional regulation is specialized RNA-binding proteins, which facilitate the interaction between sRNAs and their cognate target mRNAs.

In many bacteria, the majority of the interaction between sRNA and mRNA involves the RNA-binding protein Hfq [1, 2]. However, other functions of Hfq in regulating translation and degradation of mRNAs independently of the sRNA-mediated regulatory pathway have also been documented [3–7]. Hfq is a member of the Sm/LSm superfamily of RNA-binding proteins, which is found in almost every organism from all three domains of life [8]. These proteins have a common structural ‘core’ comprised of an N-terminal α-helix followed by a twisted five-stranded β-sheet and assemble into a variety of quaternary structures ranging from pentamers to octamers [9]. Structures of the Hfq conserved ‘core’ region from several different bacterial species are available, and in all cases a hexameric ring structure has been observed [10–14]. The conserved core of the Hfq monomer consists of an α–β_1–5_ fold, and when these pack into a hexamer, three faces are presented for interaction with RNA. The ‘proximal face’ is close to the amino-terminal of Hfq; the ‘distal face’ lies on the opposite side of the Hfq hexamer, close to the carboxyl-terminal of Hfq; and the ‘rim region’ or ‘lateral face’, which separates the proximal and distal faces, provides additional RNA-binding sites. Appended to the conserved core is a structurally disordered carboxyl-terminal domain (CTD) that is variable in size and sequence. The CTD of Hfq in *Escherichia coli* acts synergistically with the other RNA-binding faces on the conserved core and contributes to the specificity of its RNA annealing activity [15–17].

**Figure 1.**
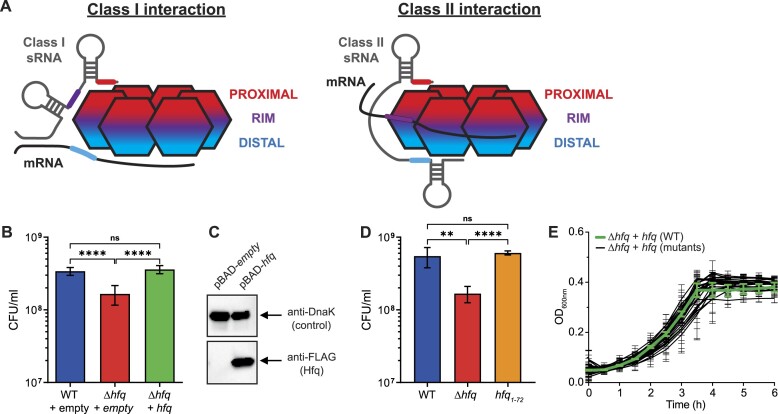
Hfq is required for survival during N starvation in *E. coli*. (**A**) Schematic showing the established mechanisms of interaction between class I and class II sRNA with Hfq and their target mRNA. Hfq monomers are represented by the six hexagons, the upper section represents the proximal RNA-binding face, the middle section represents the rim region, and the lower section represents the distal RNA-binding face. Regions of either sRNA or mRNA that associate with the different RNA-binding surfaces of Hfq are coloured according to that of the binding surfaces. (**B**) Viability of WT and Δ*hfq E. coli* expressing plasmid-borne Hfq (pBAD24-*hfq*-3xFLAG) measured by counting CFU 24 h after becoming N-starved (N-24). (**C**) Representative immunoblot of whole-cell extracts of Δ*hfq E. coli* containing either a pBAD18 empty vector control or pBAD24-*hfq*-3xFLAG, sampled at N-24. The immunoblots were probed with anti-FLAG antibody and anti-DnaK antibody (loading control). (**D**) Viability of WT and Δ*hfq E. coli*, and *E. coli* expressing Hfq with a C-terminal truncation from residues 73–102 (*hfq*_1-72_) measured by counting CFU at N-24. (**E**) Growth of Δ*hfq E. coli* expressing plasmid-borne WT or alanine mutants of Hfq (pBAD24-*hfq*-3xFLAG) under N limiting conditions. In panels (B), (D), and (E), error bars represent standard deviation (*n* = 3). In panels (B) and (D), statistical analysis was performed by Brown–Forsyth and Welch’s ANOVA (****P< 0.0001; **P< 0.01).

The biogenesis of sRNAs can occur in multiple pathways: sRNA can be made by transcription of a stand-alone non-coding gene, or can be derived by transcription starting from inside the coding region of a gene but using the same terminator [3′ untranslated region (UTR) derived], by premature transcription termination in 5′ UTR, or by processing of mRNA by ribonucleases. In all cases, interaction with Hfq is important for the stability of sRNAs and mutations in RNA interacting surfaces on Hfq can lead to decreased sRNA stability [18]. Typically, amino acid (aa) substitutions at the conserved surface-exposed proximal residue Q8, rim residues R16 and R17, and proximal face residues Y25 and K31 are widely used in many studies to understand Hfq binding to RNA. An elegant study by Schu *et al.* [18] utilized these mutants with 24 different sRNAs and their cognate mRNA partners to describe two different interaction modes of sRNA to Hfq (designated as class I and class II), which involve cooperation of the different RNA-binding surfaces on Hfq. Class I sRNAs interact with the proximal face and rim of the Hfq ring, whereas their mRNA targets bind the distal face of the ring; class II sRNAs bind to the proximal and distal faces and base pair with mRNA targets that interact with the rim (Fig. 1A) [18]. Further, Malecka *et al.* revealed that rim region and the distal face are important for compacting mRNA for optimal binding to cognate Hfq-bound sRNA [19], further underscoring that the cooperation between the RNA-binding surfaces is crucial for Hfq function.

Recently, we and others showed that in *E. coli* experiencing nitrogen (N) starvation, the Hfq-mediated post-transcriptional regulation is extensive, dynamic, and important for cell survival [20–22]. The Δ*hfq E. coli* strain does not survive or recover well from N starvation, which, in the wild-type (WT) strain, requires Hfq-mediated regulation by sRNA GlnZ [22] and SdsR [20]. Despite the central role Hfq has in sRNA stabilization and post-transcription regulation of gene expression, the involvement of individual residues of Hfq in its function has never been systematically investigated. In this study, we characterized a systematic alanine mutant library of Hfq under N starvation and provide new transcriptome-wide insights into sRNA binding and stabilization by Hfq under *in vivo* conditions when there is an increased demand for Hfq.

## Materials and methods

### Construction of Hfq mutants and bacterial growth conditions

A systematic alanine mutant library of Hfq was created by site-directed polymerase chain reaction (PCR) mutagenesis using pBAD24-*hfq*-3xFLAG [21] as the template and appropriate primers (Supplementary Data S1) and confirmed by DNA sequencing. Note that, due to the cloning procedure, an additional V aa residue was introduced at aa position 2 of Hfq so that the N-terminal aa sequence of Hfq in pBAD24-*hfq*-3xFLAG is MVAK instead of MAK. The *E. coli* strain BW25113 was used for all the experiments. The WT and Δ*hfq* BW25113 strains were obtained from the *E. coli* Genetic Stock Center. The construction of the *hfq*_1-72_ BW25113 strain is described in [21]. N starvation experiments were conducted as described in [23]. Briefly, unless otherwise stated bacteria were grown in 10 mM NH_4_Cl (for overnight cultures) or 3 mM NH_4_Cl (for N starvation experiments) in Gutnick minimal medium (33.8 mM KH_2_PO_4_, 77.5 mM K_2_HPO_4_, 5.74 mM K_2_SO_4_, 0.41 mM MgSO_4_), supplemented with Ho-LE trace elements [24], 0.4% (w/v) glucose, and 100 μg/ml of ampicillin at 37°C in a shaking (700 rpm) SPECTROstar OMEGA plate reader (BMG LABTECH) [23]. To induce expression of Hfq, l-arabinose was added at final concentration of 0.2% (w/v). Growth of bacterial cultures was either measured by optical density at 600 nm (OD_600_) over time or determined by measuring colony forming units (CFUs) from serial dilutions on lysogeny broth agar plates.

### Immunoblotting

Whole cell extracts of N-24 bacteria were prepared by pelleting bacterial cell from a 1 ml culture and resuspension in denaturing polyacrylamide gel electrophoresis (denaturing PAGE) loading buffer. Following denaturing PAGE of the sample, the gel was transferred onto polyvinylidene difluoride membrane (0.2 μm). For immunoblotting of Hfq protein, mouse monoclonal Anti-FLAG^®^ M2 antibody (Merck, F1804) was used at 1:1000 dilution. For the loading control, mouse monoclonal anti-DnaK antibody (Enzo, 8E2/2) was used at 1:1000 dilution. HRP sheep anti-mouse IgG (GE Healthcare, NA931) at 1:10 000 dilution was used as the secondary antibody. ECL Prime western blotting detection reagent (GE Healthcare, RPN2232) was used to develop the blots, which were analysed on the ChemiDoc MP imaging system and bands were quantified using Image Lab software.

### Preparation of the molecular dynamics calculations

Chain A of the *E. coli* Hfq hexamer X-ray structure (PDB: 1HK9) [25] was used to obtain the initial coordinates of the Hfq monomer in all simulations. To increase sampling efficiency, the flexible C-terminal residues (aa 66–69) were removed from the X-ray structure of Hfq [25]. The F11A, L12A, V22A, I24A, G34A, I36A, and Y55A mutants were obtained by substituting the individual residues in the WT structure. The topology and coordinate files for molecular dynamics (MD) simulations were created with the xLeap program of the AMBER 20 package [26]. To describe the protein, we used either the ff19SB or ff12SB protein force fields. The ff12SB is the earlier version of the ff14SB [27], which was, however, shown to provide superior description for some systems [28]. The ff19SB force field is the successor to both of these older force fields, showing improvements for proteins with disorder and large-scale dynamics [29]. To describe the water molecules, we have used the OPC [30] and SPC/E [31] water models in the ff19SB and ff12SB simulations, respectively, as recommended for these protein force fields [27, 29]. In all simulations, the protein was surrounded in an octahedral box of water molecules with minimal distance of 12 Å (SPC/E) or 13 Å (OPC) between the solute and the box border. Physiological ion concentration of 0.15 M was established by adding KCl ions [32] at random positions around the solute. For each system, we have performed minimization and equilibration with pmemd.MPI module of AMBER 20 according to the standard protocol [33].

### Standard MD simulations

Standard (unbiased) MD simulations were run for 5 μs, using the pmemd.cuda module and RTX 2080ti GPUs. Selected trajectories were extended up to 20 μs and multiple trajectories of each system were obtained (Table 1). The SHAKE algorithm [34] along with the hydrogen mass repartitioning [35] was applied in all simulations, allowing a 4 fs integration step. The long-range electrostatics was treated using the particle mesh Ewald methodology [36] and periodic boundary conditions were imposed to handle the system border bias. The cut-off distance for Lennard–Jones interactions was 9 Å. Langevin thermostat and Monte Carlo barostat [26] were used to maintain the temperature and pressure around 300 K and 1 bar, respectively.

**Table 1.** List of MD simulations

Mutation	Standard MD		Enhanced sampling (REST2)
	ff19SB (OPC)	ff12SB (SPC/E)	ff19SB (OPC)
WT	2 × 5 μs	2 × 5 μs	1 × 10 μs
F11A	2 × 5 μs	2 × 5 μs	1 × 10 μs
L12A	2 × 5 μs	2 × 5 μs	1 × 10 μs
V22A	2 × 5 μs	–	1 × 10 μs
I24A	1 × 20, 2 × 5 μs	2 × 5 μs	1 × 10 μs
G34A	2 × 5 μs	–	1 × 10 μs
I36A	1 × 20, 2 × 5 μs	2 × 5 μs	1 × 10 μs
Y55A	2 × 5 μs	2 × 5 μs	1 × 10 μs

En dashes in cells indicate ‘no data available’.

### Enhanced-sampling REST2 simulations

For each system, we performed REST2 (replica exchange with solute tempering) simulations [37] with 12 replicas, with the scaling factor (lambda) ranging from 1 to ∼0.6. The REST2 is a ‘brute-force’ enhanced sampling method that does not follow any particular user-defined collective variable. Instead, the accelerated sampling is achieved by modifying the potential energy function of the system, lowering the energy barriers for conformational transitions of the protein. This is coupled with the replica exchange protocol. The REST2 can be used to observe conformational changes that are beyond the timescale of standard MD simulations. The main disadvantage of this protocol is loss of the information on kinetics. In all of our REST2 simulations, the entire Hfq monomer was scaled (i.e. included in the hot zone). Only the ff19SB force field was utilized for REST2 and the CMAP potentials were scaled by the same factor as the dihedral potentials. The lambda values across the replica ladder were chosen for each system to maintain average successful exchange rate of 25%. The REST2 simulations were performed using the pmemd.cuda.MPI module of AMBER 20 and their length was 10 μs (Table 1). The simulation settings were the same as for the standard simulations (see above), except the production phase of the REST2 simulations was performed in constant volume ensemble.

### Analyses of the simulations

We used cpptraj and VMD for analysis and visualization of all MD trajectories [38, 39]. Graphs and molecular figures were prepared with gnuplot and povray, respectively. The native contacts analysis was performed with cpptraj, by first compiling a list of interatomic distances <7 Å present in the initial structure, i.e. the native contacts. These distances were subsequently evaluated at every 10th frame of all trajectories, with the native contact counted as present for distances <7 Å. Only the distances between heavy atoms of residues at least 4 aa apart in the sequence were considered for the analysis. The native contacts analysis was performed on second halves of the standard MD trajectories of each system, with multiple trajectories combined into a single ensemble. Due to slightly different overall number of native contacts in each mutant, we present percentages of the native contacts preserved rather than their absolute numbers. For the REST2 simulations, the analysis was performed on the second half of the reference (unscaled) replica of each system. The resulting data were used to calculate histograms, with bin size set to 50 and the distributions normalized. All the trajectories were extensively visually inspected.

### Hfq purification

Hfq variants were expressed from pBAD vector in Δ*hfq* TOP10 cells grown at 37°C, induced with 0.1% (w/v) l-arabinose at OD = 0.4–0.6 and harvested by centrifugation after an overnight expression at 18°C. Cells were resuspended in lysis buffer [50 mM Tris–HCl (pH 8.0), 1.5 M NaCl, 250 mM MgCl_2_, cOmplete™ EDTA-free Protease Inhibitor Cocktail (Roche)]. Resuspended cells were lysed using Emulsiflex C5 (Avestin) high-pressure homogenizer (1000 bar). Lysate was clarified by centrifugation (4°C, 37 500 × *g*, 30 min). Supernatant was incubated at 85°C for 10 min and centrifuged (20°C, 37 500 × *g*, 30 min). Resulting supernatant was incubated with ammonium sulfate (0.9 M) and centrifuged (20°C, 37 500 × *g*, 30 min). Supernatant was filtered (Sartorius Minisart^®^ 0.45 μm) and loaded on 5 ml HiTrapButyl HP (Cytiva), previously equilibrated with butyl buffer A [50 mM Tris (pH 8.0), 1.5 M NaCl, 1 M (NH_4_)_2_SO_4_]. Proteins were eluted with an isocratic gradient of butyl buffer B (50 mM Tris, pH 8.0). Fractions containing Hfq were pooled and diluted 1:5 with butyl buffer B. Pooled sample was then loaded on 5 ml HiTrap Heparin HP (Cytiva), previously equilibrated with heparin buffer A [50 mM Tris–HCl (pH 8.0), 100 mM NaCl, 100 mM KCl], and eluted with an isocratic gradient of heparin buffer B [50 mM Tris–HCl (pH 8.0), 1 M NaCl, 100 mM KCl]. Fractions containing Hfq were pooled and concentrated (Amicon^®^ Ultra-15 30 kDa, Millipore). Protein solution was loaded on Superdex 200 Increase 10/300 GL size exclusion column (S200; Cytiva) previously equilibrated with Hfq storage buffer [50 mM Tris–HCl (pH 8.0), 100 mM NaCl, 100 mM KCl, 5% glycerol].

### Isothermal titration calorimetry

Samples were buffer exchanged into isothermal titration calorimetry (ITC) buffer [20 mM Tris–HCl (pH 7.5), 100 mM NaCl, 20 mM KCl] using PD10 columns (GE Healthcare). For the U_6_ titrations, sample buffer included 5 mM MgCl_2_. Protein and RNA concentrations were measured with a NanoDrop One spectrophotometer (Thermo Fisher Scientific) using calculated extinction coefficients of 4470 M^−1^ cm^−1^ for Hfq at 280 nm, 207 400 M^−1^ cm^−1^ for A_17_ at 260 nm, and 45 360 M^−1^ cm^−1^ for U_6_ at 260 nm. ITC measurements were made at 30°C in using a MicroCal iTC200 microcalorimeter (Malvern Panalytical). Concentrations for optimal isotherms were 177 μM A_17_ in the syringe, and 12–18 μM Hfq (hexamer) in the cell. The profiles for A_17_ Hfq were corrected for the small heat of dilution of A_17_ into buffer and fitted with a single binding site model. Concentrations for optimal isotherms for U_6_ were 120 μM RNA in the syringe, and 10 μM Hfq (hexamer) in the cell. The profiles for U_6_ Hfq, corrected for comparatively small heat of dilution of U_6_ into buffer, were more complex and were fitted with a single binding model after offsetting the enthalpy change for a second, weaker binding event.

### Circular dichroism spectroscopy

Measurements were made with an Aviv 410 circular dichroism (CD) spectrometer. Spectra were measured using 1 mm pathlength quartz cuvettes. Spectra in the near-ultraviolet (near-UV) range are averages of 10 measurements, and the far-UV are averages of 5 spectra. The average spectra were normalized for sample absorbance at 280 nm.

### Crystallizations, X-ray diffraction data collection, and model refinement

Crystals were prepared by sitting droplet vapour diffusion using a mosquito crystallization robot. Crystals of pA_4_/Hfq V22A mutant appeared in 22.5% (v/v) PEGSB, 0.2 M LiSO_4_, 0.05 M zinc acetate, 0.1 M Bis-Tris (pH 7.5), where PEGSB is composed of equal parts of polyethylene glycol (PEG) 400, 550 MME, 600, 1k, 2k, 3350, 4k, 5k MME, 6k, 8k, and 10k. Crystals were treated briefly with cryoprotectant, flash frozen, and stored in liquid nitrogen. Diffraction data for A_4_/Hfq V22A were collected at Diamond Light Source station IO4 (mx33658-55) at wavelength 0.954 Å. Intensities were integrated and scaled in automated mode using xia2 (Supplementary Table S1). The structure was solved using molecular replacement the core residues 6–66 of *E. coli* Hfq, based on PDB entry 3GIB. Two Hfq hexamers occupy the asymmetric unit, leaving 38% solvent content, and the hexamers stack tightly through interactions of the proximal face and circumferential rim, but leave gaps between the stacks on the proximal face. The space in the lattice is likely filled with the N- and C-terminal tails, but these are comparatively disordered. The diffraction was anisotropic, which may reflect the disorder due to the packing gaps. One molecule of pA_4_ could be traced at the proximal face of one of the hexamers, and disordered portions of the RNA polymer could be fit at the proximal surface of the other hexamer. Anomalous fouriers did not reveal any peaks that may be due bound metal. On the distal side of the pA_4_/V22A crystal, we observe density that could fit adenines, which would represent a non-canonical interaction with pA_4_ on this surface (Supplementary Fig. S2). The electron density for the V22A/pA_4_ crystal was not satisfactory to model the details of these interactions.

The co-crystallization conditions of Hfq V22A with A_17_ were 15% (v/v) PEGSH (composed of equal parts of PEG 6k, 8k, and 10k), 0.15 M ammonium acetate, 0.1 M trisodium citrate (pH 5). Data were collected at Diamond Light Source station IO3 at wavelength 0.9763 Å. The asymmetric unit comprises one Hfq hexamer and one 17-mer RNA, with 49% solvent content. The refined map has clear density for 18 adenine bases, but only 17 were expected. The RNA is therefore likely to be averaged, with partial occupancy of 0.944, assuming complete circular permutation of the oligonucleotide over the 18 potential binding sites. This is consistent with the lack of a clear break in the connectivity of the 5′ to 3′ phosphodiester linkages. The RNA is bound on the distal face, forming the expected interactions following the known A-R-N pattern, and the exposed N bases interdigitate with a neighbouring complex related by crystallographic symmetry to form a dimeric unit through face-to-face stacking of distal faces. As found in the A_4_/Hfq V22A crystals, the A_17_/Hfq V22A molecules pack tightly into sheets but leave gaps between the sheets on the proximal face. The models of A_4_/Hfq V22A and A_17_/Hfq V22A were refined using phenix refine [40], refmac [41], and extensive model building with coot (Supplementary Table S2) [42]. The N- and C-termini are extensively disordered and could not be modelled into the electron density. A conformational ensemble was refined using MD starting from geometrically idealized termini, but this did not improve the free residual for either of the two crystals.

### RNA sequencing

Bacteria were harvested at N-24. RNA was extracted using the RNAsnap protocol [43]. Three biological replicates of each strain were taken and mixed with a phenol:ethanol (1:19) solution at a ratio of 9:1 (culture:solution) before harvesting the bacteria immediately by centrifugation. Pellets were resuspended in RNA extraction solution (18 mM ethylenediaminetetraacetic acid, 0.025% sodium dodecyl sulphate, 1% 2-mercaptoethanol, 95% formamide) and lysed at 95°C for 10 min. Cell debris was pelleted by centrifugation. RNA was purified with PureLink RNA Mini Kit extraction columns (Invitrogen, 12183018A) and largely in accordance with the manufacturer’s protocol for total transcriptome isolation except with a final ethanol concentration of 66% to increase the yield of smaller RNA species. Analysis of extracted RNA was performed following depletion of ribosomal RNA (rRNA) molecules using a commercial rRNA depletion kit for mixed bacterial samples (Lexogen, RiboCop META, #125). The ribo-depleted RNA samples were first fragmented using ultrasound (four pulses of 30 s at 4°C). Then, an oligonucleotide adapter was ligated to the 3′ end of the RNA molecules. First-strand complementary DNA (cDNA) synthesis was performed using M-MLV reverse transcriptase with the 3′ adapter as primer. After purification, the 5′ Illumina TruSeq sequencing adapter was ligated to the 3′ end of the antisense cDNA. The resulting cDNA was PCR-amplified using a high-fidelity DNA polymerase and the barcoded TruSeq libraries were pooled in approximately equimolar amounts. Sequencing of pooled libraries, spiked with PhiX control library, was performed at a minimum of 7 million reads per sample in single-ended mode with 100 cycles on the NextSeq 2000 platform (Illumina). Demultiplexed FASTQ files were generated with bcl-convert v4.2.4 (Illumina). Raw sequencing reads were subjected to quality and adapter trimming via Cutadapt [44] v2.5 using a cut-off Phred score of 20 and discarding reads without any remaining bases (parameters: –nextseq-trim = 20 -m 1 -a AGATCGGAAGAGCACACGTCTGAACTCCAGTCAC). Afterwards, all reads longer than 11 nt were aligned to the *E. coli* K12 MG1655 reference genome (RefSeq assembly accession: GCF_000005845.2) using the pipeline READemption [45] v2.0.3 with segemehl version 0.3.4 [46] and an accuracy cut-off of 95% (parameters: -l 12 -a 95). READemption gene_quanti was applied to quantify aligned reads overlapping genomic features by at least 10 nt (-o 10) on the sense strand based on RefSeq annotations [CDS (coding sequence), ncRNA (non-coding RNA), rRNA (ribosomal RNA), and tRNA (transfer RNA)] for assembly GCF_000005845.2 from 11 March 2022. Based on these counts, differential expression analysis was conducted via DESeq2 [47] version 1.24.0. Read counts were normalized by DESeq2 and fold change shrinkage was conducted by setting the parameter betaPrior to TRUE. Differential expression was assumed at adjusted *P*-value after Benjamini–Hochberg correction (*P*_adj_) < 0.05 and |log_2_FoldChange| ≥ 1. DESeq2 analysis can be found in Supplementary Data S2.

## Results

### Characterization of a systematic alanine mutant library of Hfq

Our objective was to make a systematic alanine mutant library of Hfq to identify amino acids that are important to the adaptive response to N starvation in *E. coli*. To do so, we first developed a simple experimental system to evaluate the mutant library of Hfq. Bacteria devoid of Hfq (Δ*hfq*) do not display any viability defect under N replete (N+) conditions or following short-term (20 min) N starvation [21]. However, the absence of Hfq severely compromises the viability of a population of Δ*hfq E. coli* following 24 h of N starvation (N-24) (Fig. 1B). The proportion of viable bacteria in the Δ*hfq E. coli* population can be restored to WT levels by supplying exogenous Hfq from an inducible plasmid (here l-arabinose was used as the inducer to drive transcription of *hfq* from the *araB* promoter). The plasmid-borne Hfq contained a FLAG-tag at its carboxyl terminal end to allow detection of the protein in bacterial whole-cell extracts (Fig. 1C). The Hfq of *E. coli* is 102 aa in length and residues 1–65 represent the conserved core (Supplementary Fig. S1). Given that removal of the CTD aa 73–102 does not compromise Hfq's function [48], including in its role in the adaptive response to N starvation (Fig. 1D), we systematically changed each aa residue between positions 3 and 72 to alanine (excluding the 2 alanine residues at positions 12 and 56 in the WT Hfq sequence). The mutant Hfq library consisted of 68 mutant proteins.

**Figure 2.**
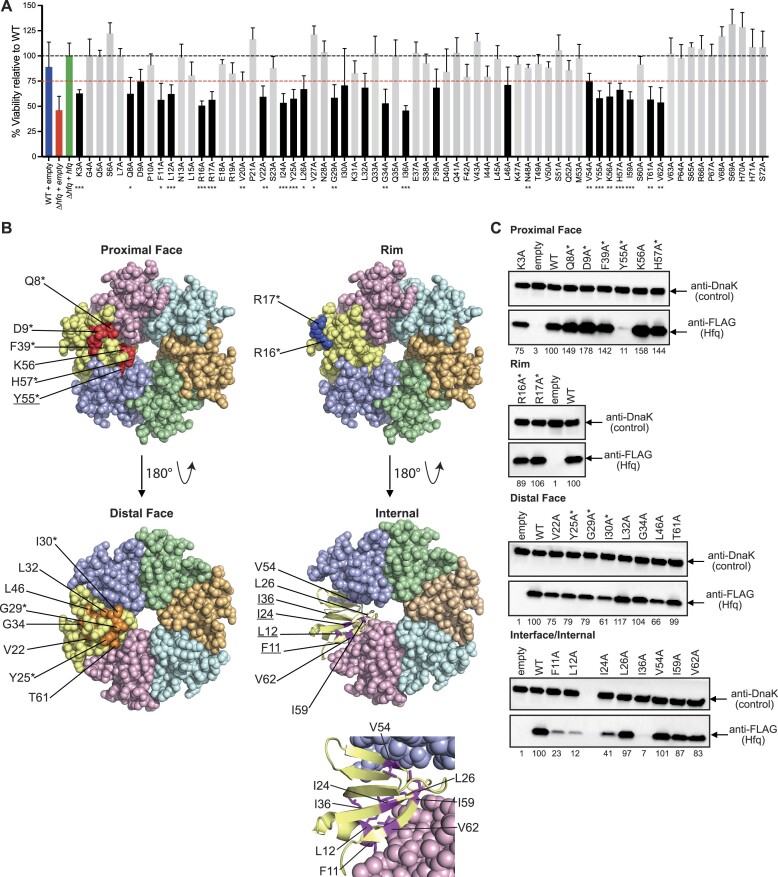
Characterization of a systematic alanine mutant library of Hfq. (**A**) Viability of WT and Δ*hfq E. coli* expressing plasmid-borne alanine mutants of Hfq (pBAD24-*hfq*-3xFLAG) measured by counting CFU following N-24. Values are represented as a percentage of viable cell counts relative to the WT complemented strain. The dashed red line indicates ≥25% drop in viable cell count. Error bars represent standard deviation (*n* = 3). Statistical analysis performed by Brown–Forsyth and Welch’s ANOVA. (***P< 0.001; **P< 0.01; *P< 0.05). (**B)**Structures of hexameric Hfq (PDB: 1HK9) with each monomer of Hfq coloured separately. The aa residues at which an alanine substitution results in ≥25% decrease in viability with respect to WT (black bars in panel A) are labelled. The aa residues at or close to the proximal face and distal face that are surface exposed (marked with *) or not fully buried within the Hfq hexamer are shown in red and orange, respectively; the surface-exposed rim residues are shown in blue; and residues fully buried and internal to the Hfq hexamer are shown in purple. Residues at which alanine substitution resulted in >50% reduction in protein expression are underlined (see panel C). Inset shows a magnified view of residues found at internally or at the monomer–monomer interfaces, with the residue sidechains shown. Note that aa K3 is not present in the Hfq structure used here. (**C**) Representative immunoblots of whole-cell extracts of Δ*hfq E. coli* containing either a pBAD18 empty vector control, or WT or alanine mutants of pBAD24-*hfq*-3xFLAG, sampled at N-24. The immunoblots were probed with anti-FLAG antibody and anti-DnaK antibody (loading control). The ratio of the intensity of bands corresponding to alanine mutants of Hfq relative to that of WT Hfq from the same immunoblot are indicated below the immunoblot.

Plasmids harbouring the Hfq mutants were introduced into Δ*hfq* bacteria, which were then grown under N replete conditions in the presence of l-arabinose. As shown in Fig. 1E, the growth dynamics of bacteria expressing the Hfq mutants did not significantly differ from that of WT bacteria. Under our conditions, growth arrest occurs when the sole N source is exhausted and for all strains this occurred ∼3.5 h following inoculation (Fig. 1E). We left the cultures until N-24 and enumerated the proportion of viable cells in the population by counting CFUs. We identified several aa residues in Hfq at which an alanine substitution adversely affected viability of Δ*hfq* bacteria (Fig. 2A). For further analyses, we selected Δ*hfq* bacteria encoding plasmid-borne Hfq mutants that displayed a ≥25% reduction (Fig. 2A, red line) in the number of viable cells in the population at N-24 compared with Δ*hfq* bacteria encoding plasmid-borne WT Hfq. Twenty-five Hfq mutants were selected (Fig. 2A, black bars). Of these 25 mutations in Hfq, as shown in Fig. 2B, 7 were surface-exposed residues at the proximal face (K3, Q8, D9, F39, H57, and Y55; note that K3 is absent from the structure shown in Fig. 2B) including the partially exposed residue K56; 5 were surface-exposed residues on the distal face (I30, G29, and Y25) including the partially exposed residues L32 and T61; 3 residues lie within the hydrophobic core (V22, G34, and L46) close to the distal face of the Hfq hexamer; 2 are surface exposed on the rim region (R16 and R17); 7 residues are located at the interface between the individual monomers of the Hfq hexamer (F11, L12, I24, L26, V54, I59, and V62); and 1 residue (I36) was internal to the Hfq monomer. We probed the cell extracts of Δ*hfq* bacteria encoding the 25 plasmid-borne Hfq mutants using anti-FLAG antibodies to determine how the expression levels of the mutant proteins differed from that of the WT protein at N-24. We quantified the intensity of the band corresponding to each mutant protein on the immunoblots and considered a mutant to be expressed to near WT level if the band intensity was within ∼75% of the band intensity of WT Hfq. Most Hfq mutants, except proximal face mutant Y55A, distal face mutant I30A, the L46A mutant within the hydrophobic core of the Hfq hexamer, several mutations (F11A, L12A, and I24A) at the interface between the individual monomers of the Hfq hexamer, and the I36A mutant that is internal to the Hfq monomer, were expressed at levels comparable to that of the WT Hfq (Fig. 2C). Thus, it is conceivable that the aa residues F11, L12, I24, I30, I36, L46, and Y55 contribute to the overall structural integrity and stability of either individual Hfq monomers or the hexamer.

### MD simulations confirm aa residues that contribute to structural integrity of the Hfq monomer

**Figure 3.**
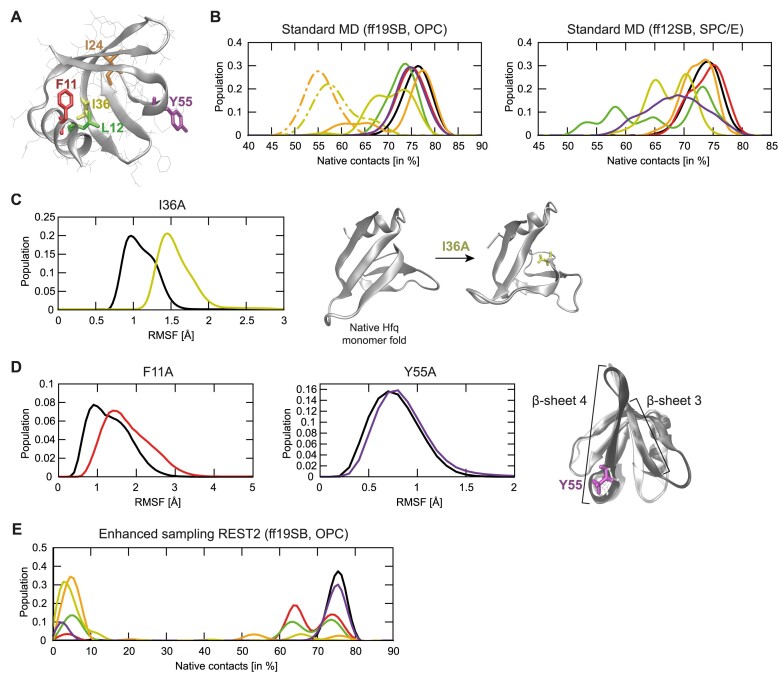
MD simulations of unstable alanine mutants of Hfq. (**A**) Structure of the Hfq monomer with the amino acids for which we studied the effects of alanine substitutions using MD simulations; the substitutions are labeled and individually coloured (F11 in red, L12 in green, I24 in orange, I36 in yellow, and Y55 in violet). The other aa residues are shown as thin grey lines. The secondary structure is indicated with grey ribbon. (**B**) Graphs of the populations of native contacts in standard MD simulations of alanine mutant Hfq protein, colour coded as in panel (A). The black line corresponds to the WT simulations. The graphs show the percentage of the interatomic contacts present in the starting structure (i.e. the native contacts) that are subsequently maintained in MD simulations of each alanine mutant of Hfq. Higher populations of fewer native contacts indicate more significant departures from the native Hfq fold. The dot–dashed lines indicate the two standard simulations that were extended to 20 μs (see Table 1). For visual clarity, the data curves were smoothed with cubic spline interpolation. (**C**) Graph of the average RMSFs of the I36A mutation (yellow curve) and comparison with the WT (black curve) in standard MD simulations using ff19SB. The collapse of the native Hfq protein fold with I36A mutation as observed by MD simulations is shown. The mutation causes structural collapse of the native fold during which the β-sheet surface splits open. Very similar changes were also observed with the L12A and I24A mutations (not shown). (**D**) Graphs as in panel (C) but of the α-helix (F11A, red) and β-sheet residues (Y55A, violet) and their comparison with the WT (black curve) in standard MD simulations using ff19SB. For Y55A, shown in silver is the starting structure and in black the structure after the β-sheets 3 and 4 have split; indicated in purple is the Y55 residue. (**E**) Graph as in panel (B) but of the native contacts in the reference replica of the REST2 enhanced sampling MD simulations.

MD simulation allows the conformational space available to macromolecules to be explored and is an effective method for investigating structural changes induced by mutations. To investigate alanine substitutions at aa residues suspected to be important for the structural integrity of the Hfq monomer, we conducted standard MD simulations on Hfq monomer using two protein force fields (Table 1; see also the ‘Materials and methods’ section for difference between ff12SB and ff19SB protein force fields, which are models used to calculate the potential energy and forces between atoms to simulate their interactions). We chose Hfq mutants, which appeared to be most structurally destabilized in Fig. 2C (F11A, L12A, I24A, I36A, and Y55A) for MD analysis (Fig. 3A). Briefly, MD plots in Fig. 3B reveal the percentage of interatomic contacts present in the starting structure (i.e. the native contacts), which are maintained through the course of the MD simulation. Higher populations of fewer native contacts indicate greater departure from the native protein fold. The results revealed visible signs of instability of the native structure in the mutant proteins, which were particularly prominent for the L12A, I24A, and I36A mutants (Fig. 3B). For example, in the I36A mutant, which is an internal residue in the Hfq monomer, the presence of alanine creates a void for which the protein compensates with structural rearrangements, causing the native fold to split open and fall apart (Fig. 3C). However, the structural basis of the disruptive influence of the surface-exposed F11A and Y55A mutation was more elusive. By measuring the root mean square fluctuation (RMSF), which is a measure of fluctuations in a proteins secondary structure during MD simulation, we observed alternative docking arrangements of the α-helix 1 to the rest of the domain and generally increased flexibility in the F11A mutant (Fig. 3D). The Y55A mutation led to increased flexibility of the nearby β-sheets as well, eventually causing the β-sheets 3 and 4 to split open (Fig. 3D). Overall, all the mutants selected for MD analysis were generally less stable in standard MD simulations than the WT Hfq protein. However, due to limited sampling, we did not observe the loss of the protein structure beyond the early stages of the unfolding process. Even extending the standard MD simulations up to 20 μs was insufficient to observe complete loss of the characteristic Hfq fold (Fig. 3B and Table 1). Therefore, we performed enhanced sampling REST2 simulations, where we could observe virtually complete loss of the native Hfq fold for the mutants selected for analysis (Fig. 3E). The only exception was the Y55A mutant that was still relatively stable by the simulation end but still less so compared with the WT protein. Importantly, the WT Hfq protein always retained ∼75% of native contacts in both standard MD and REST2 simulations. Since no restraints or artificial biases were at any point applied to reinforce the native structure, the MD analysis supports the view that the alanine substitutions at aa residues F11, L12, I24, I36, and Y55 severely disrupt the Hfq monomer structure. Lastly, we note both the ff12SB and ff19SB protein force fields revealed only minor differences within the limit of the sampling and the same qualitative trends in terms of stability of the mutants (Fig. 3B). The exception seems to be the Y55A mutant that was significantly less stable with ff12SB than with ff19SB (Fig. 3B). In general, the ff19SB maintained slightly more native contacts but the observed differences are very minor. The specific water models (OPC versus SPC/E) utilized with each force field could also be the source of these differences.

### Alanine substitutions at conserved residue V22 or G34 have modest effects on RNA-binding affinity and structural integrity of Hfq

**Figure 4.**
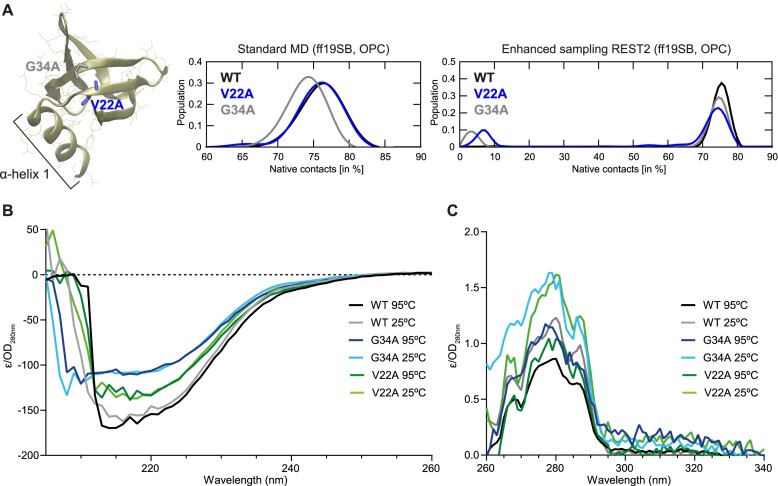
Properties of V22A and G34A alanine mutants of Hfq. (**A**) Structure of the Hfq monomer indicating aa residues V22 and G34; α-helix 1 is indicated. Graphs of the native contacts in standard (ff19SB, OPC) and REST2 enhanced sampling (ff19SB, OPC) MD simulations of alanine mutant Hfq protein (as in Fig. 3B and E). The black line corresponds to the WT simulations, with the blue and grey lines corresponding to the V22A and G34A Hfq alanine mutants, respectively. The graphs indicate how many of the interatomic contacts present in the experimental WT structure (i.e. the native contacts) are subsequently maintained in MD simulations of each system. Higher populations of fewer native contacts indicate more serious departures from the native Hfq fold. (**B**) CD analysis of WT, V22A, and G34A alanine mutants of Hfq protein, at 25°C and 95°C between wavelengths in the range of 205–260 nm. (**C**) Same as in panel (B), but between wavelengths in the range of 260–340 nm.

Of the Hfq mutants that displayed compromised viability at N-24, but were expressed to near or at WT levels (18 in total), several [13] have been previously implicated to be important for, or involved in, RNA binding by Hfq [48–50]. These are K3, Q8, D9, R16, R17, Y25, G29, L32, F39, K56, I59, H57, and T61 (Figs 1A and 2A). To the best of our knowledge, residues V22, G34, L26, V54, and V62 have not been previously implicated in Hfq function. V22 and G34 are within the hydrophobic core of Hfq monomer close to the distal face, and residues L26, V54, and V62 are at the monomer–monomer interface. Alanine substitutions at the monomer–monomer interface could affect the conformation of Hfq hexamer, rendering it functionally ineffective during N starvation. As residues V22 and G34 are fully conserved in Hfq from different bacteria (Supplementary Fig. S1) and located in the hydrophobic core of Hfq, we focused our analysis on investigating why alanine substitution at these positions compromised Hfq function under N starvation. Standard MD simulation of both V22A and G34A Hfq mutants revealed no signs of instability of the native structure and similar number of native contacts were maintained by both mutants as in the WT Hfq monomer (Fig. 4A). We note subtle differences in the native contacts maintained between the G34A and the WT Hfq monomer (Fig. 4A). Under enhanced MD simulation, the V22A and G34A seem to lower the monomer stability, but not to a degree observed for the other mutants (Fig. 4A).

CD spectroscopy in the UV and near-UV regions provides insights into protein secondary and tertiary structure, respectively, and temperature-dependent changes in the spectra can inform on relative stability. The CD spectra in the UV range of the WT and V22A and G34A Hfq mutants revealed little changes between 25°C and 95°C, indicating that all proteins are highly stable (Fig. 4B). Further, in the near-UV range, we observed that spectra of both mutants are similar and characteristic of that of the WT protein at 25°C and that comparable changes occur in all three proteins with heating to 95°C, indicating a comparable level of tertiary structure stability (Fig. 4C). However, we do note small differences when comparing the spectra of each protein (Fig. 4B), possibly indicating subtle changes in the secondary structure between the mutants and between the mutants and the WT Hfq (Fig. 4C), consistent with the MD simulations (Fig. 4A). Collectively, the CD spectroscopy analysis shows that alanine substitution at either position V22 or G34 do not cause large-scale structural perturbations or instability in Hfq.

The so-called A-R-N motif (where A is adenosine, R a purine, and N any nucleotide), found in upstream regions of those mRNAs that are regulated by class I sRNAs, specifically interacts with the distal face of Hfq. The proximal face preferentially binds to U-rich RNA sequences, present at the 3′ end of most sRNAs. To study the RNA-binding activities of both mutants, we measured the affinities of V22A, G34A, and WT proteins to a poly(A) RNA (A_17_) and poly(U) RNA (U_6_) by ITC. We found that the V22A Hfq mutant bound to the A_17_ RNA roughly 2.5–5 times more tightly than the WT protein (Table 2). No such marked difference in the affinity for the V22A and WT protein was found for binding poly(U) RNA (U_6_), which prefers the proximal face of Hfq. However, the affinity of the G34A mutant was ∼2 times tighter for U_6_ than the WT protein. Taken together, the results indicate that the affinity of the distal face for RNA is altered in both mutant proteins, and the alanine substitution at G34 further alters the affinity for RNA of the proximal face.

**Table 2.** Interactions of Hfq and RNA measured by ITC

Protein:RNA interaction	*K*_d_ (nM)	*N* (RNA/Hfq hexamer)	Δ*H* (kcal/mol)	Δ*S* (cal/mol/°C)	Replicates
WT Hfq:A_17_	51.5 ± 18.0	1.31 ± 0.10	−25.3 ± 0.1	−50.1 ± 2.8	4
V22A Hfq:A_17_	10.5 ± 3.4	1.43 ± 0.19	−28.5 ± 0.4	−57.0 ± 11.5	3
G34A Hfq:A_17_	26.3 ± 13.2	0.75 ± 0.11	−26.4 ± 0.6	−52.3 ± 3.0	2^a^
WT Hfq:U_6_	42.2 ± 12.8	0.96 ± 0.07	−12.6 ± 0.1	−7.6 ± 2.8	3
V22A Hfq:U_6_	56.6 ± 7.5	1.02 ± 0.04	−11.5 ± 0.1	−5.0 ± 0.4	3
G34A Hfq:U_6_	19.3 ± 8.3	0.83 ± 0.01	−15.9 ± 0.2	−16.9 ± 1.4	3

^a^Errors are based on repeated measurements are standard errors, except for the G34A mutant with A_17_, where the errors are based on spread.

To further elaborate on the properties of V22A Hfq mutant, we obtained diffracting co-crystals of the V22A mutant using two poly(A) RNAs, pA_4_ and A_17_. The crystals of V22A/pA_4_ diffract to roughly 3.0 Å, and the crystals of V22A/A_17_ diffract to 2.1 Å. The structures were solved using molecular replacement of the core residues 6–66 of *E. coli* Hfq, based on PDB entry 3GIB [12]. For the V22A/pA_4_ crystal, two Hfq hexamers occupy the asymmetric unit, leaving 38% solvent content, and the hexamers stack tightly through interactions of the distal face and circumferential rim, but leave gaps between the stacks on the proximal face (Supplementary Fig. S2). For the V22A/A_17_ crystal, a hexamer occupies the asymmetric unit with 49% solvent content, and the lattice interactions are like those observed for the V22A/pA_4_ crystal, including the gap between proximal face stacks. These are unusual lattice organizations, and the space in both lattices is likely filled densely with the N- and C-terminal tails of Hfq, but these are comparatively disordered and could not be modelled. The gap spacing is likely maintained through weak, multivalent interactions and may be a balance of attractive and electrostatically repulsive interactions, in analogy to liquid–liquid phase separations.

**Figure 5.**
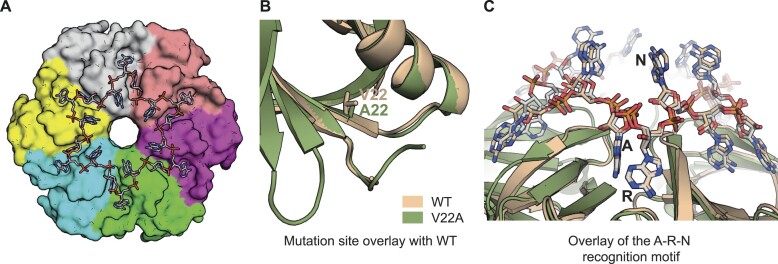
Crystal structure of *E. coli* Hfq V22A in complex with A_17_. (**A**) 3D structure of V22A Hfq, with one A_17_ molecule making canonical A-R-N interactions. (**B**) Overlay of the mutant and WT subunit with zoom onto the site of mutation, revealing little conformational change. (**C**) Overlay of the mutant and WT subunit showing the similarity of the interaction with the A-R-N recognition motif. The N nucleotide differs in conformation, most likely as a result of the crystal lattice packing. PDB: 9H45.

One molecule of pA_4_ RNA could be traced at the distal face of one of the hexamers, and disordered portions of the RNA polymer could be fit at the distal surface elsewhere on the hexamer (Supplementary Fig. S2). For the V22A/A_17_ crystal, clearly defined density could be resolved of the RNA (Fig. 5A). An overlay of the WT and V22A Hfq mutant crystal structures reveal similar conformations around the site of substitution, within the error of the intermediate resolution of the crystal structure of the mutant protein (Fig. 5B). Focussing on the interaction of the distal face with the A_17_ RNA, we found that the interactions of the V22A Hfq mutant are very close to those seen for the canonical A-R-N pattern recognition that has been well characterized by earlier structural studies for the WT protein (Fig. 5C). Overall, we conclude that alanine substitutions at V22 or G34, while increasing affinity for A_17_ and U_6_, respectively (Table 2), may only modestly alter the overall structural conformation and distal face RNA-binding interface of Hfq.

### The transcriptomes of V22A and G34A mutants resemble that of bacteria devoid of Hfq

**Figure 6.**
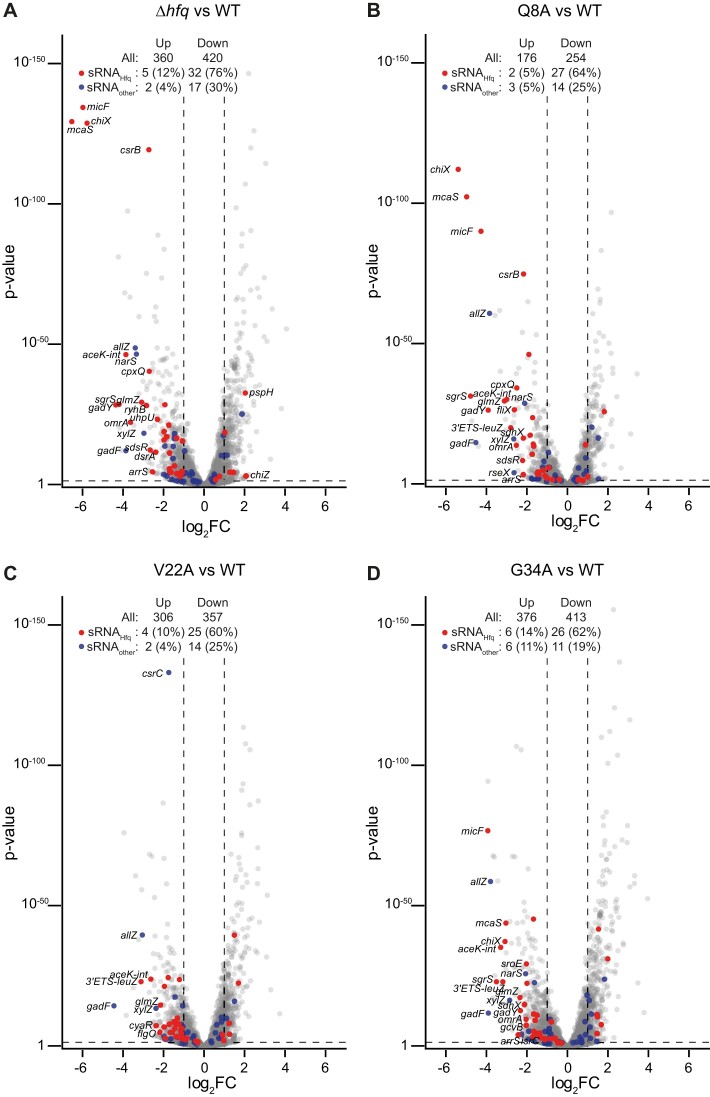
Alanine substitution at Hfq aa residues V22 or G34 lead to substantial changes in the transcriptome. (**A**) Volcano plots of differential RNA abundance in the transcriptome of Δ*hfq E. coli* versus Δ*hfq E. coli* expressing plasmid-borne WT Hfq. (**B**) Same as in panel (A) but Δ*hfq E. coli* expressing plasmid-borne Q8A Hfq versus Δ*hfq E. coli* expressing plasmid-borne WT Hfq. (**C**) Same as in panel (A) but Δ*hfq E. coli* expressing plasmid-borne V22A Hfq versus Δ*hfq E. coli* expressing plasmid-borne WT Hfq. (**D**) Same as in panel (A) but Δ*hfq E. coli* expressing plasmid-borne G34A Hfq versus Δ*hfq E. coli* expressing plasmid-borne WT Hfq. Analysis was performed by DESeq2. sRNAs that have been previously shown to interact with Hfq during N starvation conditions (sRNA_Hfq_) are shown in red, and other sRNAs and ncRNAs (sRNA_other_) are shown in blue. sRNA and ncRNA differentially expressed more than 2 log_2_ (i.e. a >4-fold change) are labelled. The number and percentage (of total detected) of differentially expressed genes, sRNA_Hfq_ and sRNA_other_ are indicated (log_2_FC > 2 and *P*-value < 0.05).

**Figure 7.**
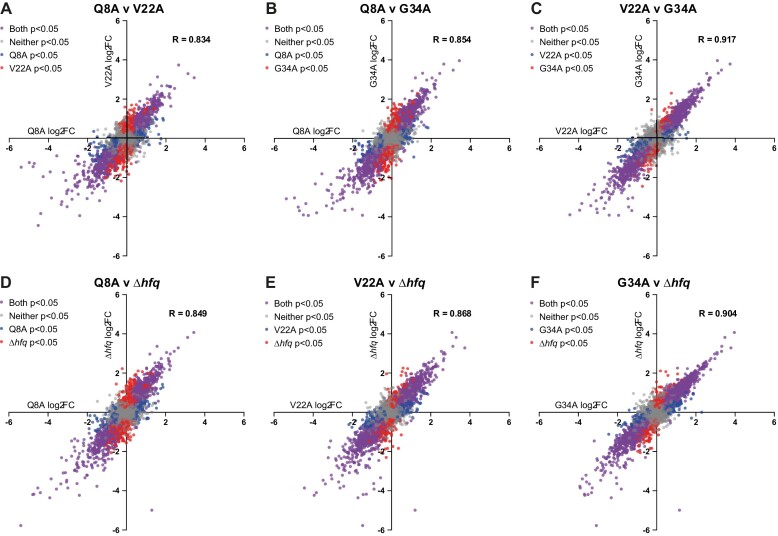
Perturbations to the transcriptome of bacteria expressing alanine mutants of Hfq largely correlate. Differential gene expression, relative to WT, was compared between bacteria either lacking Hfq, or containing different mutants of Hfq. Scatterplots comparing log_2_FC of individual genes in Δ*hfq E. coli* or Δ*hfq E. coli* expressing plasmid-borne Q8A, V22A, and G34A alanine mutants of Hfq (log_2_FC relative to Δ*hfq E. coli* expressing plasmid-borne WT Hfq) are shown. Genes found to be differentially expressed with respect to bacteria expressing plasmid-borne WT Hfq with a *P*-value < 0.05 in both pairwise comparisons are shown in purple; genes found to be differentially expressed relative to bacteria expressing plasmid-borne WT Hfq in just one of the compared strains are indicated in blue or red (see inset key for each scatterplot); genes found not to be differentially regulated relative to bacteria expressing plasmid-borne WT Hfq are shown in grey. Pearson correlation coefficient (*R*) for each comparison is shown.

We considered that the increased affinity of the V22A and G34A mutants for RNA (Table 2) could compromise the kinetics of sRNA and mRNA interactions needed to maintain homeostasis in post-transcriptional regulation of gene expression during N starvation, when there is an increased demand for Hfq [20]. Therefore, we compared the transcriptomes of bacteria expressing WT Hfq with that of bacteria containing the V22A and G34A Hfq mutants at N-24. Surface-exposed residue Q8, at the proximal face of Hfq, is important for Hfq-mediated post-transcriptional regulation involving both class I and class II sRNAs *in vivo*. However, the Q8A Hfq mutant, unlike the V22A or G34A Hfq mutants, does not display any altered affinity for poly-A or poly-U RNA compared with the WT protein [49]. Thus, we also obtained the transcriptomes of N-24 bacteria expressing the Q8A Hfq mutant and bacteria devoid of Hfq (Δ*hfq*) as controls to evaluate the extent of perturbation in the transcriptome of N-24 bacteria containing the V22A and G34A Hfq mutants. We defined differentially expressed genes as those with expression levels changed ≥2-fold with a false discovery rate adjusted *P*< 0.05. As shown in Fig. 6A–D, the expression of 306/6.7% of total genes and 376/8.3% of total genes were upregulated, and 357/7.9% of total genes and 413/9.1% of total genes were downregulated in bacteria expressing V22A and G34A alanine mutants of Hfq, respectively (Fig. 6A–D), comparable to the number dysregulated genes in bacteria devoid of *hfq* (360/7.9% of total genes up and 420/9.2% of total genes down). Put differently, the extent of perturbation to the transcriptome in bacteria encoding the V22A or G34A Hfq mutant is comparable to that in the transcriptomes of bacteria expressing the Q8A Hfq mutant or entirely devoid of Hfq (Δ*hfq*). In further support of this view, we next compared the degree to which genes were differentially expressed between the different strains. Correlation analysis of the fold change of the differentially expressed genes in the transcriptomes of bacteria containing the Q8A, V22A, G34A, and Δ*hfq* with each other revealed that the direction in which the transcriptomes are perturbed did not substantially differ (Fig. 7). Put simply, broadly the same genes were differentially regulated in the transcriptomes of all bacteria regardless of which aa residue was mutated and to a similar extent as in the transcriptome of Δ*hfq* bacteria. It thus seems that an alanine substitution at V22 or G34 in Hfq has a comparable impact on gene expression during N starvation as in bacteria encoding the Q8A Hfq mutation or devoid of Hfq.

### The transcriptomes of V22A and G34A mutants reveal destabilization of both class I and class II sRNA transcripts

**Figure 8.**
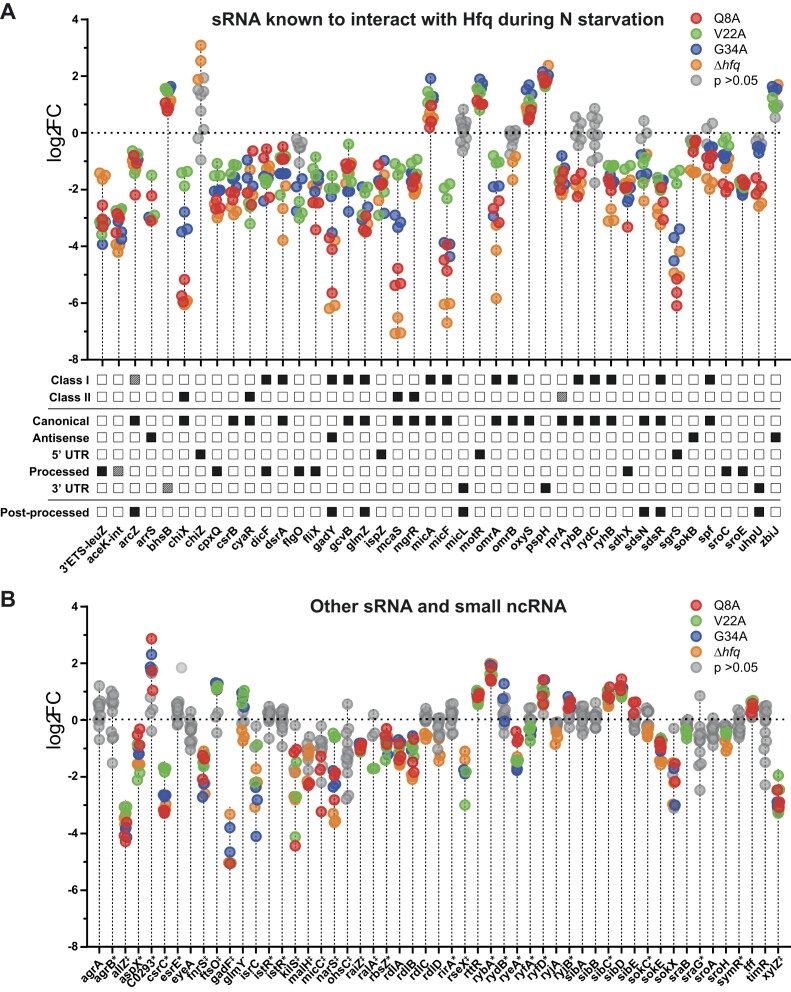
Alanine substitution at Hfq aa residues V22 or G34 leads to indiscriminate destabilization of Hfq-associated sRNA. (**A**) Dot plot showing log_2_FC of individual sRNA previously shown to interact with Hfq during N starvation in Δ*hfq E. coli* and Δ*hfq E. coli* expressing plasmid-borne Q8A, V22A, and G34A alanine mutants of Hfq, all relative to Δ*hfq E. coli* expressing plasmid-borne WT Hfq. Results where an sRNA was not found to be differentially expressed with a *P*-value < 0.05 by DESeq2 in a given strain are shown in grey. The sRNA class (where experimentally established), the nature of their biogenesis, and whether the sRNA undergoes processing following biogenesis are shown below the dot plot. Dashed squares indicate cases where there is uncertainty whether the given sRNA displays the property listed. (**B**) Same as in panel (A) but for sRNA and ncRNA not detected to interact with Hfq during N starvation. The symbol ^‡^ Indicates sRNA known to interact with Hfq, but not detected to interact during N starvation; the symbol * indicates sRNA that do not interact with Hfq.

As Hfq mediates most of its regulatory function by stabilizing sRNAs and facilitating their binding to their cognate target RNA transcripts, we focused on the expression levels of sRNAs in the transcriptomes of bacteria encoding the V22A and G34 to better understand their role in Hfq function. From the literature, we expected that both class I and class II sRNAs to be destabilized in the transcriptome of the Q8A mutant (recall that the proximal face of Hfq is required for binding to both classes of sRNAs) and in the Δ*hfq* strain. As shown in Fig. 8A, we noted that both class I and class II sRNA transcripts that are known to interact with Hfq during N starvation [20], were downregulated in the context of the V22A or G34A mutants. Put differently, we noted that the same Hfq interacting sRNA transcripts were downregulated in bacteria containing the V22A or G34A Hfq mutant as in bacteria containing the Q8A Hfq mutant (and Δ*hfq* mutant), which is known to be compromised for both classes of sRNA binding *in vivo* [18]. However, this was not the case for non-coding RNA transcripts that do not interact with Hfq during N starvation (sRNA known to interact with Hfq, but not during N starvation are marked with ‘‡’, and sRNA and other non-coding RNA that do not interact with Hfq at all are marked with ‘*’) (Fig. 8B), underscoring the pivotal role Hfq has in sRNA stabilization. We did not observe any correlation between how the sRNAs are made, or whether they are further processed following transcription, with the degree of their downregulation (Fig. 8A). However, the extent by which some of the sRNA transcripts were downregulated substantially differed. For example, the class II sRNA ChiX is downregulated by ∼42- and ∼54-fold in the bacteria containing the Q8A mutation or devoid of Hfq, respectively, but only by ∼3- and ∼8-fold in bacteria containing the V22A and G34A mutation, respectively (Fig. 8A). Further, some sRNAs such as ChiX, MicF, and McaS are more negatively affected in the bacteria expressing the G34A Hfq mutant than in bacteria expressing the V22A Hfq mutant, suggesting that alanine substitution at V22 and G34 affects Hfq function somewhat differently. In sum, the results reveal that an alanine substitution at residues V22 or G34 in the hydrophobic core of Hfq, close to the distal face, leads to the destabilization of both class I and class II sRNAs under *in vivo* conditions.

## Discussion

Hfq is central to the post-transcriptional regulation of gene expression in diverse bacterial species. Much of our understanding of structure–function relationships in Hfq has come from reductionist *in vitro* analyses involving a limited number of prototypical sRNAs and their cognate RNA partners. However, emerging data from genome-wide mapping of the RNA species that interact with Hfq *in vivo* [2, 51–55], have revealed that Hfq can facilitate several hundred RNA–RNA interactions, with the complexity of Hfq-mediated RNA–RNA interaction networks compounded by differing expression levels and binding affinities of different RNA species for each other and Hfq. Our current understanding of how Hfq interacts with RNA is based on many *in vitro* biochemical, biophysical, and structural studies (see e.g. [10, 14, 18, 19, 48, 50, 56–58]). These have revealed that surface-exposed conserved aa residues on three faces on the toroidal structure of the Hfq hexamer (proximal, distal, and rim) cooperatively contribute to bind different species of RNA. However, whilst these *in vitro* studies have provided important mechanistic details into Hfq-mediated regulation of RNA, they do not capture the inherent complexity of the cellular system. Hence, as highlighted by this study, some molecular details of Hfq-dependent regulation of gene expression remained elusive. We have revealed that conserved residues V22 and G34 within the hydrophobic core, close to the distal face of the Hfq hexamer, have a profound impact on Hfq function. Alanine substitution at conserved residue V22 or G34 have a modest adverse effect the structural integrity, and the 3D structure of the V22A Hfq mutant in complex with RNA was found to be largely unchanged relative to the WT protein. Surprisingly, alanine substitution at conserved residue V22 or G34 displayed increased affinity for RNA via the distal and proximal (only G34A) face. However, strikingly, bacteria expressing either the V22A or G34A Hfq mutant displayed transcriptome-wide destabilization of sRNAs to a similar extent seen in Q8A mutant or Δ*hfq* bacteria. This, consequently, led to global dysregulation of gene expression, resembling that seen in bacteria devoid of Hfq, suggesting the modest defects seen with the mutant proteins *in vitro* are amplified in the cellular context. Notably, both class I and II sRNA transcripts are destabilized in bacteria expressing either the V22A or G34A Hfq mutant, suggesting that alanine substitution at either V22 or G34 adversely impacts both the proximal (impacting both class I and class II) and distal (impacting class II sRNAs) RNA-binding faces of Hfq. Indeed, V22 and G34 pack into a hydrophobic pocket and an alanine substitution could impact on the presentation of the α-helix 1 containing Q8 (Fig. 4A), which contributes to both class I and class II sRNA interaction at the proximal face of Hfq. We propose that this hydrophobic pocket could be targeted for the rational design of compounds to interfere with the RNA-binding activity of Hfq.

In previous work, we reported that the relative sRNA abundance increases over time under N starvation, whereas Hfq levels remain relatively consistent [21, 23, 59]. The Hfq-mediated RNA–RNA interaction network, under a given condition, likely exists in an equilibrium state where individual Hfq–RNA–RNA interactions are in a constant state of flux depending on the transcript levels and relative binding affinities of the different RNA species, with the equilibrium state shifting when the abundance of sRNA changes. This equilibrium of Hfq–RNA–RNA interactions likely has an important role in maintaining homeostasis in the post-transcriptional control of genetic information flow, to a degree we still do not fully understand. We propose that any subtle changes (in this case increase) in affinity for RNA resulting from the V22A or G34A mutation become exacerbated in the cellular context where competition for Hfq occupancy is high. This, consequently, could compromise the equilibrium of Hfq–RNA–RNA interactions and lead to large-scale perturbation of gene expression, akin to that seen in bacteria devoid of Hfq.

Hfq’s contribution to regulating bacterial gene expression extends beyond facilitating RNA–RNA interactions or conferring sRNA stability, and includes the biogenesis of ribosomes [60] and tRNA modification [61]. Therefore, perturbations to Hfq function will also have an indirect impact on the cellular proteome. Indeed, we note that the proteome of N-24 bacteria devoid Hfq is substantially perturbed compared with that of WT bacteria (Supplementary Fig. S3). It is possible that subtle conformational changes to the hydrophobic pocket near the distal face of Hfq into which V22 and G34 pack influence how Hfq interacts with itself to form oligomers, or with the RNA degradosome [62] or Hfq regulators, such as HqbA [63]; these interactions could affect the proteome indirectly. Andrade *et al.* [60] reported that mutations in the distal face of Hfq adversely impacted maturation of 16S rRNA. However, inspection of the 16S and 23S rRNA peaks of the RNA samples prepared for the transcriptome experiment do not reveal anything unusual in the mutant samples (not shown). The detailed study of how individual mutations in Hfq impacts the proteome will be the subject of future research.

In sum, this study underscores the value of using systems level analysis to probe the structure–function relationships of global, nucleic acid-binding regulators of gene expression, such as Hfq. We have shown that an increase in such a protein’s affinity for nucleic acid substrate(s) can have profound adverse consequences in the cellular context, providing novel insights into the protein’s functions. Such analyses thus have the potential to identify unknown Achilles’s heels within nucleic acid-binding proteins for the rational design of, in this case, antibacterial compounds, which may elude *in vitro* analyses.

## Supplementary Material

gkaf019_Supplemental_Files

## Data Availability

The RNA-seq discussed in this publication is accessible through ArrayExpress: E-MTAB-14054. The proteomics data discussed in this publication can be accessed through PRIDE: PXD045656. The crystal structure of *E. coli* Hfq V22A in complex with A_4_ and A_17_ can be accessed through Protein Data Bank (PDB): 9GUS and 9H45, respectively.
