# Supplementary material for: Transcriptome-scale analysis uncovers conserved residues in the hydrophobic core of the bacterial RNA chaperone Hfq required for small regulatory RNA stability
Source: Nucleic Acids Res. 2025 Jan 27;53(3):gkaf019. doi: 10.1093/nar/gkaf019 (PMC11770335; doi:10.1093/nar/gkaf019)
Supplement: gkaf019_Supplemental_Files [file gkaf019_supplemental_files.zip › Supplementary Figures.docx]

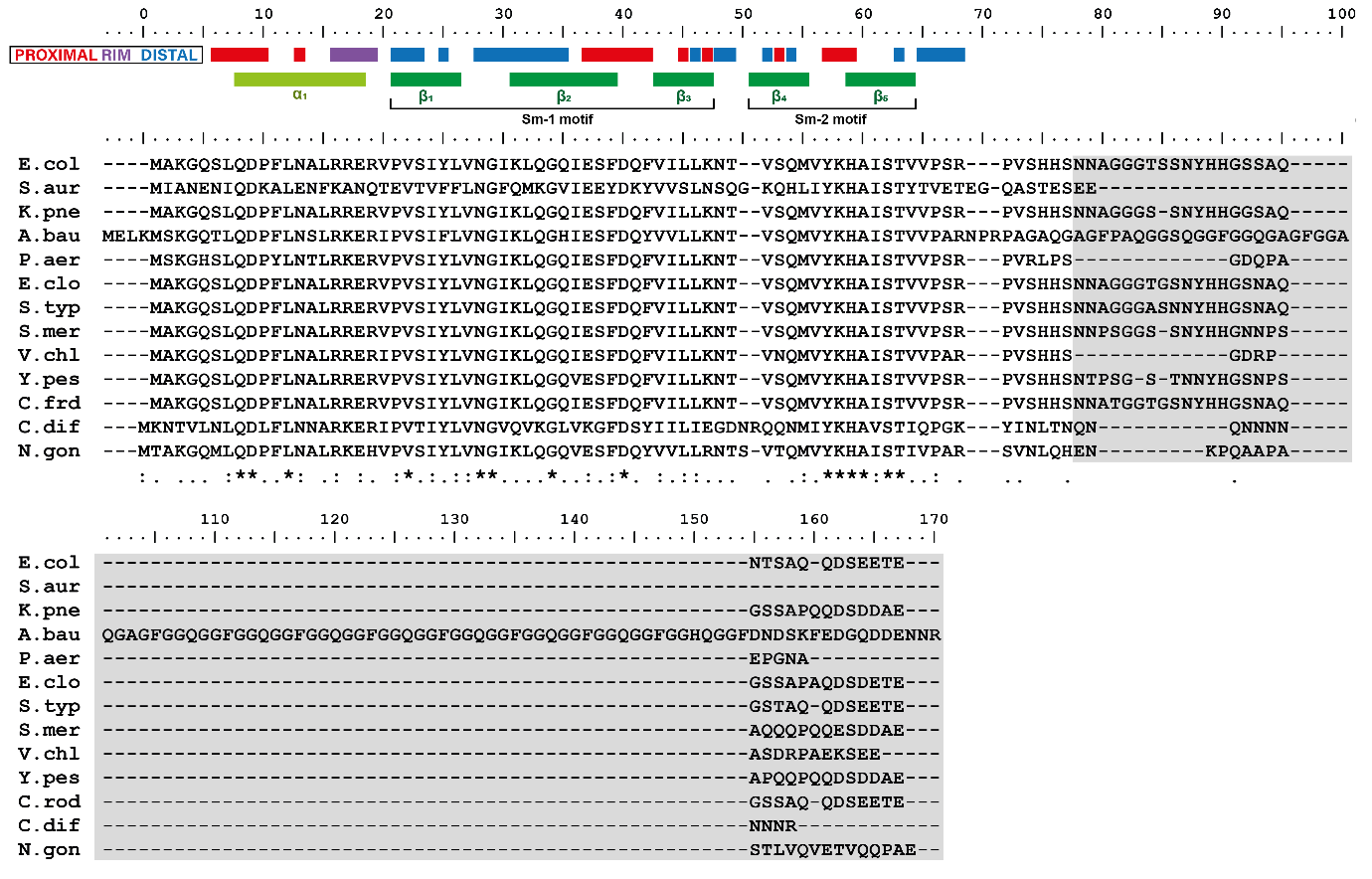


**Supplementary Figure 1.**

Alignment of Hfq amongst clinically relevant bacterial species. Fully, partially, and poorly conserved residues are indicated below the alignment with * : and . respectively. Residues found at the proximal, rim and distal RNA-binding faces are indicated with red, purple and blue respectively. α-helix, β-sheets, and Sm-like fold motifs are also indicated. Residues highlighted in grey correspond to the disordered C-terminal tail. Abbreviations correspond to the following species: E.col, *Escherichia coli* K12-MG1655; S.aur, *Staphylococcus aureus* NCTC 8325; K.pne, *Klebsiella pneumoniae* HS11286; A.bau, *Acinetobacter baumannii* ATCC 19606; P.aer, *Pseudomonas aeruginosa* PAO1; E.clo, *Enterobacter cloacae* ATCC 13047; S.typ, *Salmonella typhi* CT18; S.mer, *Serratia marcescens* Db11; V.chl, *Vibrio cholera* 10432-62; Y.pes, *Yersinia pestis* D182038; C.frd, *Citrobacter freundii* CFNIH01; C.dif, *Clostridioides difficile* 630; N.gon, *Neisseria gonorrhoeae* FA1090.


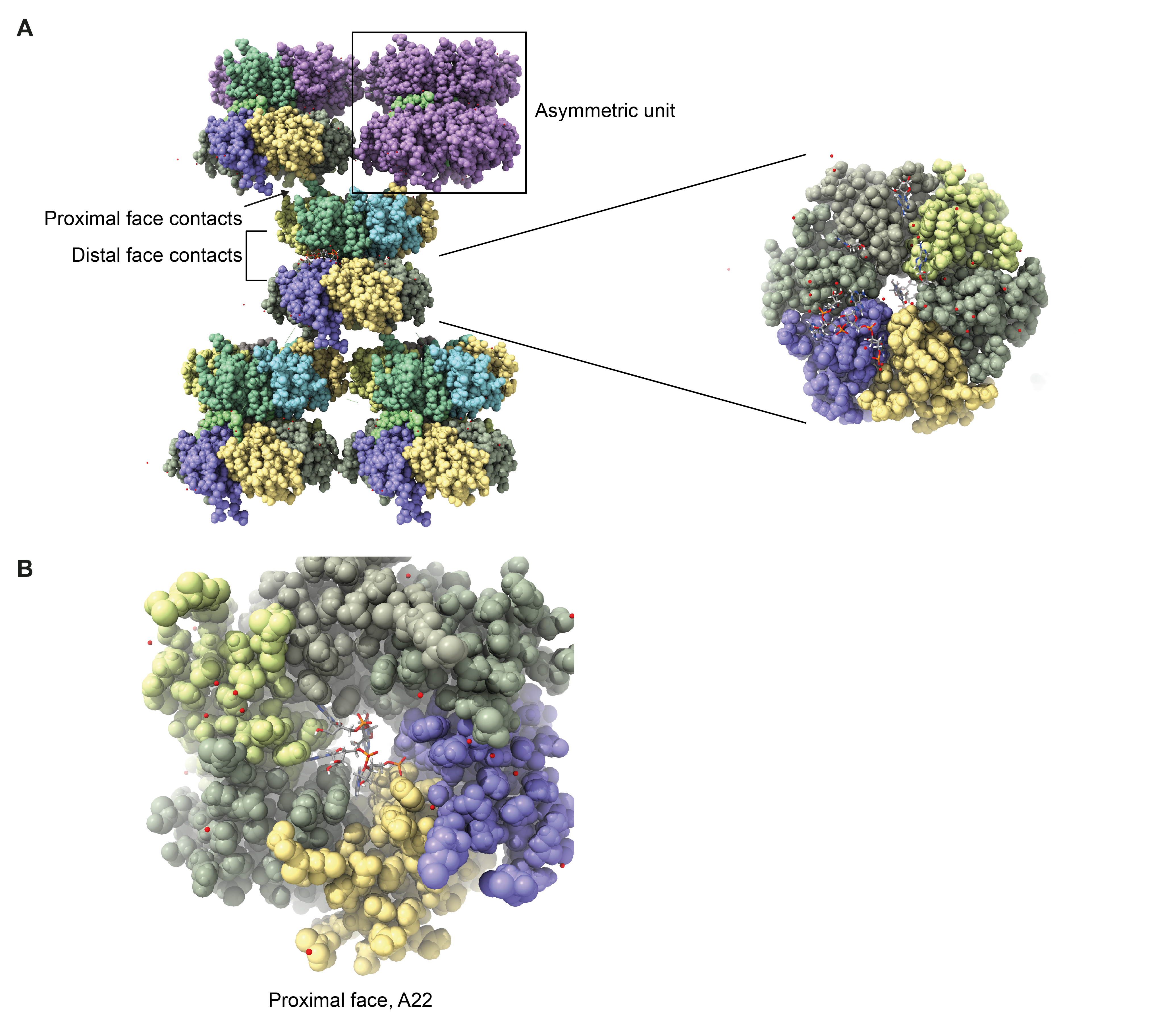


**Supplementary Figure 2.**

**(A)** The crystal lattice organisation of V22A Hfq pA_4_. Two hexamers of Hfq occupy the asymmetric unit. The two hexamers stack through interactions of the distal face, and in the perpendicular directions, through the circumferential rim. The distal interfacial contacts include shared interactions with RNA, and the panel on the right shows the view into the distal face of the lower of the two hexamers, with one pA_4_ molecule making canonical A-R-N interactions. Adenines are seen with partial occupancy at other sites of the distal face, but the lattice contacts may disfavour their formation. The hole in the centre reveals an adenine that is interacting with the proximal face in a non-canonical interaction mode. In contrast to the distal face packing, the proximal face has a gap that is occupied by disordered regions of the Hfq termini (typically 1-5 and 67 to 102). These appear to form a densely packing interface with high degree of disorder and might resemble the ensemble conformations associated with liquid phase separation state. **(B)** The interactions of A with the proximal surface. The electron density, which is not well resolved, has approximate three fold symmetry and is likely to represent an average of six orientations of the base. Other adenine bases may partially occupy part of the pockets that are preferred binding sites for uracil bases.


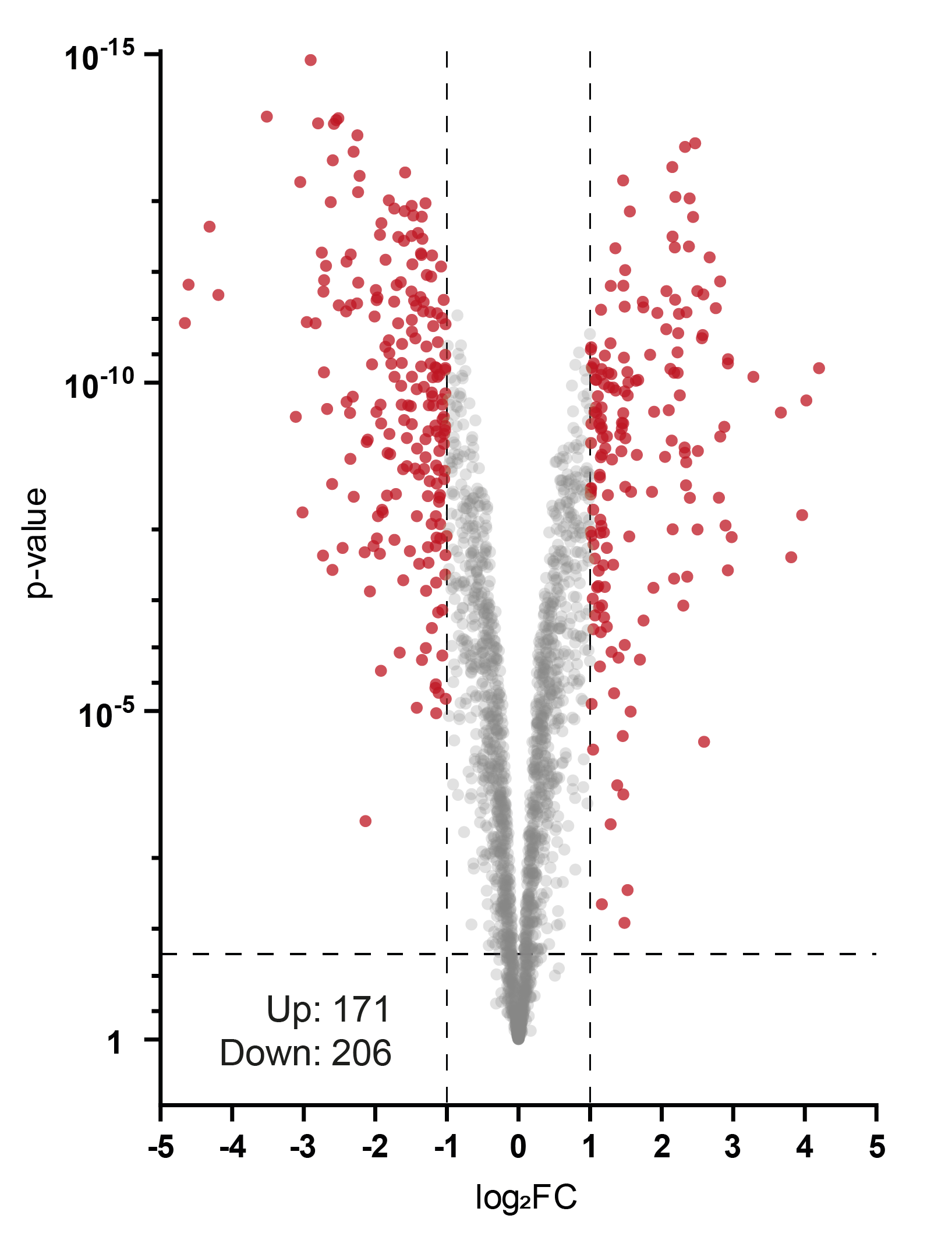


**Supplementary Figure 3.**

Volcano plot of differential protein levels in N-24 Δ*hfq* bacteria shown as a log_2_ change from wild-type bacteria. Proteins differentially expressed more than 1 log_2_ (i.e., a greater than 2-fold change) are coloured red. The number of differentially expressed proteins are indicated (log_2_FC > 1 and p-value <0.05).

**Supplementary Table 1. Crystallography Parameters**

| **Crystal** | **pA_4_/Hfq V22A (9GUS)** | | | **A_17_/Hfq V22A (9H45)** | | |
| --- | --- | --- | --- | --- | --- | --- |
|  | **Overall** | **Low** | **High** | **Overall** | **Low** | **High** |
| **High resolution limit, A** | 2.64 | 7.15 | 2.64 | 2.08 | 5.64 | 2.08 |
| **Low resolution limit, A** | 63.07 | 63.09 | 2.68 | 57.66 | 57.68 | 2.12 |
| **Completeness** | 98.8 | 99.9 | 96.5 | 99.1 | 91.8 | 99.7 |
| **Multiplicity** | 3.6 | 3.6 | 3.7 | 6.9 | 6.4 | 6.6 |
| **I/sigma** | 6 | 32.8 | 0.5 | 3.3 | 9.7 | 1.5 |
| **Rmerge (I)** | 0.231 | 0.094 | 2.319 | 0.509 | 0.133 | 2.025 |
| **Rmerge (I+/-)** | 0.183 | 0.049 | 1.893 | 0.469 | 0.123 | 1.895 |
| **Rmeas (I)** | 0.272 | 0.11 | 2.713 | 0.551 | 0.144 | 2.196 |
| **Rmeas (I+/-)** | 0.258 | 0.069 | 2.677 | 0.553 | 0.145 | 2.254 |
| **Rpim (I)** | 0.142 | 0.057 | 1.402 | 0.209 | 0.056 | 0.841 |
| **Rpim (I+/-)** | 0.182 | 0.049 | 1.893 | 0.292 | 0.076 | 1.208 |
| **CC half** | 0.759 | 0.529 | 0.336 | 0.968 | 0.991 | 0.246 |
| **Wilson B factor** | 36.53 | 36.53 | 36.53 | 12.25 | 12.25 | 12.25 |
| **Anomalous completeness** | 98.1 | 97.5 | 95.7 | 98 | 88.3 | 98.5 |
| **Anomalous multiplicity** | 1.8 | 1.8 | 1.9 | 3.5 | 3.5 | 3.4 |
| **Anomalous correlation** | 0.939 | 0.995 | 0.027 | 0.023 | -0.103 | -0.071 |
| **Anomalous slope** | 0.935 | 0.935 | 0.935 | 0.958 | 0.958 | 0.958 |
| **dF/F** | 0.216 | 0.216 | 0.216 | 0.23 | 0.23 | 0.23 |
| **dI/s (dI)** | 0.863 | 0.863 | 0.863 | 0.777 | 0.777 | 0.777 |
| **Total observations** | 107257 | 5382 | 5439 | 258949 | 11536 | 12714 |
| **Total unique** | 29610 | 1507 | 1471 | 37712 | 1805 | 1912 |
| **Assuming spacegroup** | P 1 | | | C2 | | |
| **Unit cell** | 69.182  69.212  73.193  63.54  89.30  60.04 | | | 116.250  67.150  84.580  90.00  104.64  90.00 | | |
| **a:**  **b:**  **c:**  **alpha:**  **beta:**  **gamma:** |  |  |  |  |  |  |

**Supplementary Table 2. Crystallography data collection and refinement statistics** (Statistics for the highest-resolution shell are shown in parentheses).

| **Crystal (PDB code)** | **pA_4_/Hfq V22A (9GUS)** | **A_17_/Hfq V22A (9H45)** |
| --- | --- | --- |
| **Resolution range**, A | 57.28 - 2.9 (3.004 - 2.9) | 57.66 - 2.08 (2.120 - 2.080) |
| **Space group** | P 1 | C2 |
| **Unit cell**  **a:**  **b:**  **c:**  **alpha:**  **beta:**  **gamma:** | 69.182  69.212  73.193  63.54  89.30  60.04 | 116.250  67.150  84.580  90.00  104.64  90.00 |
| **Unique reflections** | 29610 (1471) | 37712 (1912) |
| **Completeness (%)** | 98.1 (95.7) | 99.1 (99.7) |
| **Wilson B-factor** | 57.67 | 17.05 |
| **Reflections used in refinement** | 22236 (2224) | 37680 |
| **Reflections used for R-free** | 10240 | 1879 (158) |
| **R-work** | 0.3064 (0.4127) | 0.274 (0.342) |
| **R-free** | 0.3642 (0.4235) | 0.323 (0.401) |
| **Number of non-hydrogen atoms** | 6256 | 3703 |
| **- Macromolecules** | 6218 | 3421 |
| **- Ligands** | 1 | 0 |
| **- Solvent** | 37 | 282 |
| **Protein residues** | 786 | 380 |
| **RMS (bonds)** | 0.013 | 0.003 |
| **RMS (angles)** | 1.65 | 0.679 |
| **Ramachandran favored (%)** | 87.14 | 93.35 |
| **Ramachandran allowed (%)** | 11.15 | 5.37 |
| **Ramachandran outliers (%)** | 1.71 | 1.28 |
| **Rotamer outliers (%)** | 9.36 | 4.21 |
| **Clashscore** | 25 | 19.96 |
| **Average B-factor** | 68.73 | 28.54 |
| **- Macromolecules** | 68.68 | 29.72 |
| **- Solvent** | 68.25 | 24.93 |
